# Ferrocene-Modified Polyelectrolyte Film-Coated Electrode and Its Application in Glucose Detection

**DOI:** 10.3390/polym11030551

**Published:** 2019-03-22

**Authors:** Zhiping Jiang, Yonggang Shangguan, Qiang Zheng

**Affiliations:** MOE Key Laboratory of Macromolecular Synthesis and Functionalization, Department of Polymer Science and Engineering, Zhejiang University, Hangzhou 310027, China; zhiping1120@zju.edu.cn (Z.J.); zhengqiang@zju.edu.cn (Q.Z.)

**Keywords:** ferrocene, biosensor, redox polymer, polyelectrolytes

## Abstract

A polyelectrolyte film-coated electrode for the quantitative detection of glucose was reported. Carbon nanotubes, graphene oxide and polyelectrolyte with a ferrocenyl group were used to modify an enzyme electrode to facilitate the electron transfer between glucose oxidase and the electrode. Cyclic voltammetry and amperometric methods were adopted to investigate the effects of different polyelectrolytes and carbon nanomaterials on the electrochemical properties of enzyme electrodes. The results indicate that the ferrocenyl groups on a polyelectrolyte skeleton act as a mediator between the redox center of glucose oxidase and the electrode, which efficiently enhances the electron transfer between a glassy carbon electrode and glucose oxidase. The calibration curve of the sensor shows a linear range from 0.2 to 5 mM for glucose response. The sensor can achieve 95% of the steady-state current within 10 s. The electrodes also present high operational stability and long-term storage stability.

## 1. Introduction

A simple, accurate and quick determination method of trace glucose is significantly important. As such, various glucose sensors have been developed in recent years and applied in many fields, including clinical chemistry [1,2,3,4,5], the food and beverage industry [6,7,8,9], environmental monitoring [10,11,12,13] and the military [14,15,16,17]. Among the diverse glucose sensors reported, the glucose oxidase (GOx)-based glucose biosensor is a widely used commercial sensor that has undergone tremendous development due to its excellent performance [18,19]; however, there are still some problems to be improved such as the sensitivity, responsiveness and stability in its electrical signal. The GOx biosensor conventionally uses oxygen as the electron transfer mediator and indirectly measures glucose by evaluating the oxygen consumption or the amount of the H_2_O_2_ formation in the substrate catalysis process with GOx [20]. However, such an approach is easily affected by the disturbance from electroactive substances such as ascorbic acid [21] and the fluctuation of the oxygen concentration in the environment [22]. Therefore, to overcome these shortcomings, many electron transfer mediator-based GOx biosensors have attracted extensive attention [23,24,25]. Usually, substances chosen as electron transfer mediators should meet the following requirements: Good electrochemical reversibility (corresponding to a fast electron-transfer rate), low redox potentials (corresponding to little disturbance from electroactive substances), good stability, low solubility in water and good resistance to oxygen.

Ferrocene (Fc) has a sandwich-shape molecular structure and is a highly rich electronic system with strong aromaticity. Besides the above advantages, it presents good heat stability, high reactivity, easy structural modifications and so forth. In addition, the molecular dimension of ferrocene matches the GOx active center and it can transfer electrons through a rapid redox reaction. Therefore, ferrocene and its derivatives are considered as ideal redox mediators in the construction of mediator-based biosensors [26,27,28]. However, ferrocene in the oxidation state can dissolve in water and has thus leads to the instability of the electrode, so some pioneering work has been reported to improve the above issues. Tajik et al. [29] adopted a new type of ferrocene derivative compound to replace ferrocene, enabling it to strongly adhere to a graphene paste electrode surface. Karan [30] fabricated a stable amperometric enzyme electrode by mixing GOx and ferrocene-modified silica gel particles with a carbon paste matrix, which was packed into an electrode holder. Deng et al. [11] used the direct attachment of the ferrocene-based mediators onto polymeric films to prevent the mediator from leaching. However, there still exist some shortcomings, such as the instability of GOx and the far distance between the electron transfer complex and the redox center.

The preparation of biosensors aims to immobilize enzymes on the surface of the electrode, making the GOx active center as close to the electrode as possible so that the substrate and reaction products can pass through the enzyme layer in enzymatic reaction. The sol–gel method is a commonly used enzyme immobilization technology. With the stabilization of the crosslinking agent, a polyelectrolyte film provides a three-dimensional network suited for the encapsulation of enzymes, reducing the loss of enzymes and preventing the electrode from degrading.

**Scheme 1:**
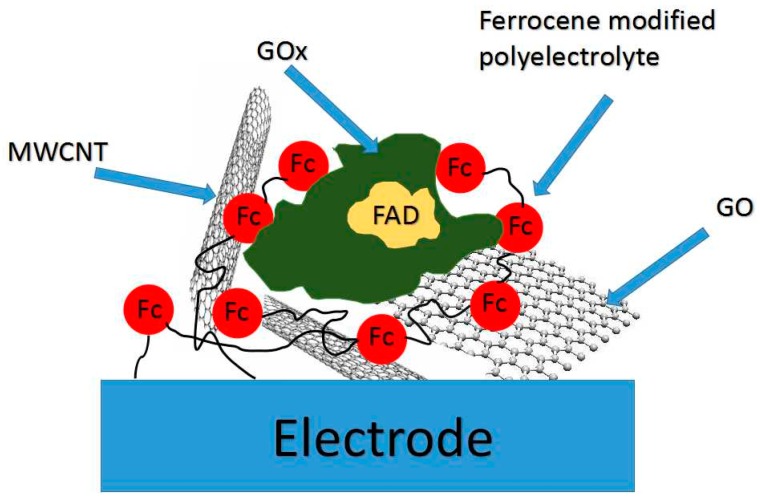
Schematic diagram of enzyme electrode structure.

Recently, our group found that Fc-modified polyelectrolyte could aggregate in water via a rapid hydrophobic association mechanism to form a stable redox solution [31,32,33]. In this work, we report different synthesized Fc-modified polyelectrolytes for enhanced electrochemical glucose biosensing, including polyacrylic acid, polyethyleneimine and polyacrylamide. With the immobilization of glucose oxidase (GOx) into the cross-linked Fc-modified polyelectrolyte network structure, a polyelectrolyte film-coated electrode was prepared, as seen in Scheme 1. The resulting biocomposite displays high sensitivity, a wide detection range and better stability towards glucose detection. In addition, graphene and carbon nanotubes were also introduced because their higher conductivity and surface area may increase the performance of the modified electrode.

## 2. Materials and Methods

### 2.1. Materials

Polyacrylic acid (PAA, *M_w_* = 1.0 × 10^5^), polyethyleneimine (PEI, *M_w_* = 2.5 × 10^4^, 99%), glucose oxidase (1.6 × 10^5^ U/g), triethylamine (Et_3_N), ethylenediamine, sodium hydroxide, potassium hydroxide, boric acid, potassium chloride, ethylene glycol diglycidyl ether (EGDGE) and sodium hypochlorite aqueous solution were purchased from Aladdin Inc. (Shanghai, China). Benzotriazol-1-yl-oxytripyrrolidinophosphonium hexafluorophosphate (PyBOP), ferrocene-carboxylic acid D(+)-glucose and dialytic tubes (molecular weight cut-off, MWCO = 1.4 × 10^4^) were obtained from Sinopharm Chemical Reagent Inc. (Shanghai, China). Multi-walled carbon nanotubes (MWCNT, with a purity above 95 wt%, prepared by chemical vapor deposition) with an average diameter of 30–50 nm and a length in the range of 10–20 mm were purchased from Shenzhen Nano-Technologies Port Co., Ltd., China. Water used for the preparation of the aqueous solutions (except for NMR measurements) was purified with a water purification system (ULUPURE) unless stated otherwise. A stock solution of 1 M glucose was prepared using deionized water and subjected to mutarotation at room temperature for 24 h. All the reagents were used without further purification.

### 2.2. Preparation of the Enzyme Electrode

The schematics for the preparation of PAA-Fc, PEI-Fc and polyacrylamide(PAM)-Fc are presented in Schemes S1–S3 in the Supplementary Material, respectively. PAA-Fc was synthesized through slightly modified procedures based on those published by Harada et al. [34], while PEI-Fc and PAM-Fc were synthesized according to the published procedures [31,32,33].

Graphite oxide was synthesized from graphite powder using Hummers’ method. One milligram/milliliter of graphene oxide (GO) aqueous dispersion was prepared by ultrasonicating for 60 min and subsequently centrifuging for 10 min at 3000 rpm. The MWCNT/GO hybrid composite was prepared as reported elsewhere [24]. Fresh GOx (6 mg/mL) was added into the hybrid composite and then subjected to sonication, which was able to facilitate the homogeneous dispersion of GOx inside the MWCNT/GO hybrid composite. Finally, the hybrid biocomposite was centrifuged at 1500 rpm to remove the loosely bound MWCNT and GOx.

Prior to modification, the bare glassy carbon electrode (GCE) was polished to a mirror-like surface with 1, 0.3 and 0.05 μm alumina slurry, respectively. It was rinsed thoroughly with deionized water between each polishing step. The electrode was successively sonicated in the mixed solution (nitric acid and acetone with a mass ratio of 1:1) and deionized water, then allowed to dry under N_2_. The redox film on the modified electrode was made by mixing the following solutions in order, as shown in Figure S4. First, 14 μL of a polyelectrolyte solution (10 mg/mL in H_2_O) and 3 μL of hybrid biocomposite were mixed by vortexing. Then, 0.75 μL of an aqueous EGDGE (10%, *v*/*v*) solution was added and vortexed. Finally, the GCE surface was cast with 5 μL GOx hybrid biocomposite suspension and dried in the atmosphere for at least 18 h at room temperature to fabricate the GO/GCE.

### 2.3. Electrochemical Measurements

A conventional three-electrode system consisting of a glassy carbon working electrode, a Ag/AgCl/3M KCl reference electrode and a platinum wire counter electrode was adopted in this work. A measuring cell with total volume of 10 mL was used. Cyclic voltammograms (CV) were recorded with a CHI 440 electrochemical workstation (CH Instruments, Austin, TX, USA) connected to a computer. Deaeration of the solution was performed by purging pure nitrogen for at least 20 min before the test. For amperometric measurements, the electrode system was set to +250 mV versus Ag/AgCl/3 M KCl while stirring the solution gently. A 0.10 M phosphate-buffered solution (PBS, pH 7.2, Na_2_HPO_4_/NaH_2_PO_4_ system) containing 0.7 wt% NaCl was used as the electrolyte solution.

## 3. Results and Discussion

### 3.1. Electrochemical Properties of the Ferrocene-Modified Polyelectrolyte Solution

The transfer efficiency of the redox polymer communicating with the redox centers of enzymes is dependent upon the polymer type. Previously, three kinds of polyelectrolyte solutions were investigated electrochemically in order to understand the sensing principle and determine their validity in biosensors applications. In this work, the solution was initially tested in phosphate-buffered saline (PBS) at pH 7.2 since this is a commonly used background electrolyte for these applications. Considering that ferrocene groups have the characteristics of reversible single-electron redox reactions, cyclic voltammetry (CV) was carried out to evaluate the redox property of the polymer. The analysis of the redox kinetics can also provide information about the electron transfer kinetics and the electroactive surface area of the electrode.

**Figure 1:**
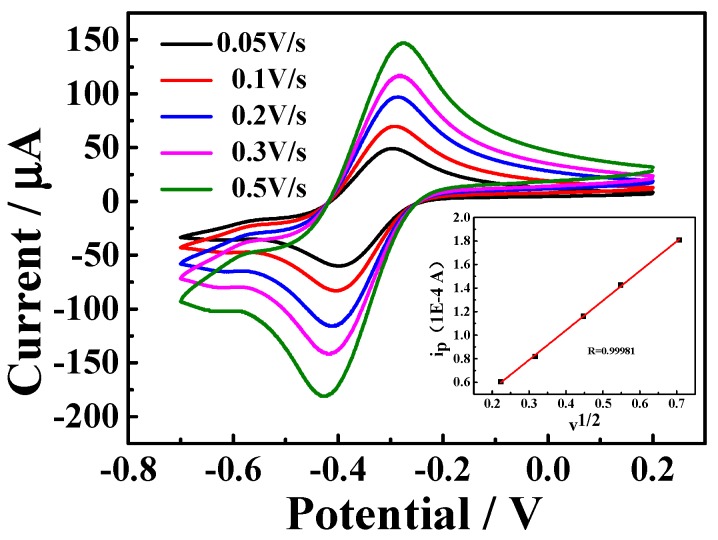
Cyclic voltammograms of 2 wt% polyacrylic acid (PAA)-ferrocene (Fc) in phosphate-buffered saline (PBS) solution under different scan rates (from 0.05 to 0.5 V/s). Inset: Plot of oxidation peak current and reduction peak current vs. square root of scan rate.

Figure 1 gives the CV curves of the PAA-Fc solution at different scan rates. The PAA-Fc sample presents a closed and reversible curve with an obvious redox peak. Correspondingly, no redox peak was found in the PAA solution due to the absence of redox-active groups. These results indicate that Fc was successfully grafted on the PAA. According to the characteristics of the CV, a half peak method was used to obtain the peak potential and current. As seen in Figure 1, the peak current ratio of oxidation and reduction is about 1 (ip1/ip2 ≈ 1), indicating that the electrochemical reaction of PAA-Fc in PBS is stable and reversible. According to electrochemical theory, the electrochemical reactions on the electrode surface can be divided into diffusion control and surface adsorption control. As for electrode reactions dominated by diffusion, the peak current increases linearly with the square root of the scan rate (ip ~ *λ*^1/2^ being a straight line), while it increases linearly with the scan rate for that controlled by surface adsorption (ip ~ *λ* is a straight line). The inset in Figure 1 indicates that the reduction of ferrocene is a typical diffusion-controlled process on the electrode surface. (The results of PAM and PEI also confirm it as described below).

The effect of concentrations on the electrochemical behavior of the PAA-Fc solution were also studied (Figure S5). A shift in peak potential appears for the 2% PAA-Fc aqueous solution. In view of the results shown in Figure 1, this indicates that the shift in the peak potential of the 2% PAA-Fc aqueous solution is common for different scan rates and glucose concentrations. It may be ascribed to the nature change of the 2% PAA-Fc aqueous solution, because the concentration of this PAA-Fc solution is higher than the critical association concentration (~0.5 wt%) and leads to the formation of an association structure. The ferrocene hydrophobic groups gather due to hydrophobic association and thus an easier oxidation of ferrocene occurs. As a result, a negative shift in oxidation potential takes place. However, PAA is negatively charged and largely surrounds around ferrocene groups, making gathered ferrocene groups difficult to reduce. Therefore, a higher reduction potential for ferrocene appears. In addition, it can be seen from the inset that the peak current increases with the increase of the PAA-Fc concentration in a good linear relationship. A pair of well-defined redox peaks is also observed for PAM-Fc and PEI-Fc (Figure S6), suggesting that PAM-Fc and PEI-Fc can also be used to modify enzyme electrodes to accelerate the electron transfer between electrodes and enzyme active sites. However, differing from the PAA-Fc solution, another oxidation peak is found in the PAM-Fc and PEI-Fc solution, perhaps because of the dissociation of amino and the difficulty of ionization.

### 3.2. Effects of the Type of Polyelectrolyte on the Electrode Performance

**Figure 2:**
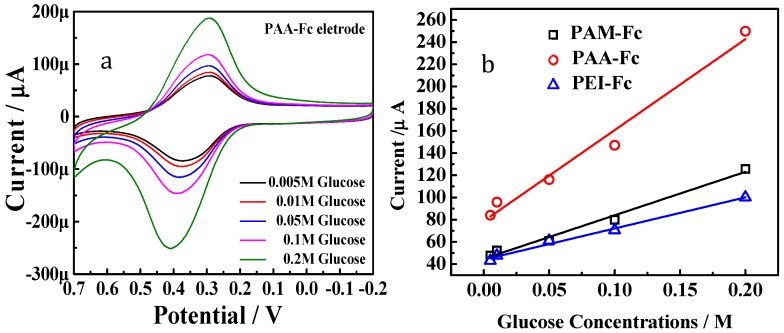
Typical cyclic voltammograms of PAA-Fc-modified electrodes under different glucose concentrations (**a**) and the plot of the oxidation peak current vs. the glucose concentration with PAA-Fc, polyethyleneimine (PEI)-Fc and PAM-Fc modified electrodes (**b**).

To investigate the effect of polyelectrolyte type on the performance of electrodes, the cyclic voltammograms of the electrodes were measured in the presence of different glucose concentrations. Figure 2a gives the CV curves of various glucose samples with different concentrations detected through the enzyme electrode modified by PAA-Fc. It can be seen that the enzyme electrodes modified by PAA-Fc present good sensitivity for all glucose samples under the concentration range investigated, differing from the case of blank electrodes (shown in Figure S7). This indicates that there is a relatively good linear relationship between the response current and glucose concentration, which can be detected quantificationally [35]. Furthermore, Figure 2b gives a comparison of CV curves of glucose samples detected through different enzyme electrodes modified by PAA-Fc, PAM-Fc and PEI-Fc, respectively. It was found that all these samples appear to have excellent sensitivity to glucose. However, the glucose samples with different concentrations can present distinct response currents for these modified electrodes and the enzyme electrode modified by PAA-Fc has a greater response current than those modified by PAM-Fc and PEI-Fc. This may be because the negatively charged PAA-Fc provides a good combination with the positively-charged glucose oxidase, which thus accelerates the electron transfer between the electrode and enzyme.

### 3.3. Effects of Carbon Nanomaterials on the Modified Electrode

The electrochemical behaviors of GOx/CNT/PAA-Fc- and GOx/CNT-GO/PAA-Fc-modified electrodes were investigated to understand the influence of GO on the response sensitivity of the modified electrode surface (Figure S8). Both GOx/CNT/PAA-Fc and GOx/CNT-GO/PAA-Fc electrodes present a pair of reversible redox peaks, but the GOx/CNT-GO/PAA-Fc electrode shows an obvious increase in peak current due to the coating of GO. As expected, after the addition of GO sheets into the composite film, the oxidation peak currents of the GOx/CNT-GO/PAA-Fc-modified electrode increase (curves b and b’), which is consistent with previous results [24]. This may be due to the fact that doped GO greatly increases the number of contact sites between the matrix film and GOx, which subsequently facilitates the electron shuttle between the mediator and electrode. In addition, both the GOx/CNT/PAA-Fc or GOx/CNT-GO/PAA-Fc electrode present an enhancement of the oxidation current peak in the presence of 3.0 mmol/L glucose, which means that both GOx/CNT/PAA-Fc and GOx/CNT-GO/PAA-Fc electrodes have good electrocatalytic activity with glucose.

For the hybridized film containing the GO-modified electrode, both the roughness and conductivity increase. In this work, the film was used as the electronic transfer channel to improve the transfer efficiency between Fc and GOx and consequently the electrocatalytic activities of the electrode was enhanced. In addition, the film makes it easier for enzymes to be immobilized due to the existence of more active sites. It is noted that the redox peak potential moves to a higher potential in the presence of glucose. These results indicate that the PAA-Fc film formed on the electrode surface partly impedes the adsorption and diffusion of glucose on the surface of GOx, which clearly influences the electrocatalytic properties of GOx.

**Figure 3:**
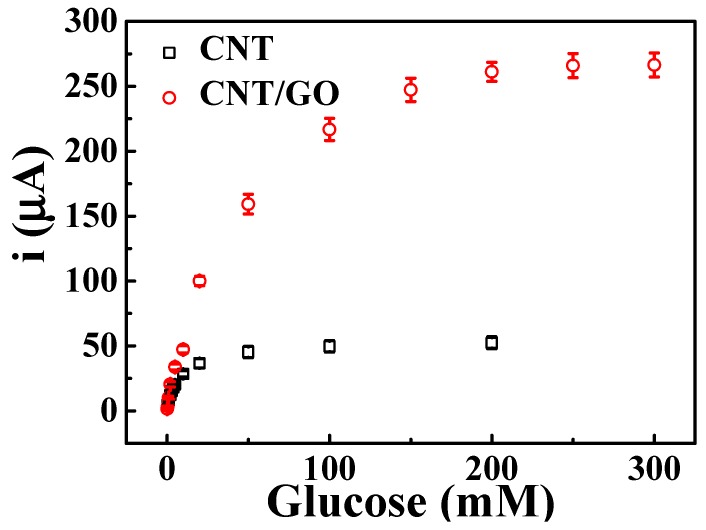
Dependence of the current response of the two enzyme electrodes on the glucose concentration.

The electrode synchronously modified by carbon nanotubes and graphene oxide was compared with that of the electrode modified only by carbon nanotubes. It was found that the carbon nanotube-modified electrode had a lower response current to glucose and can achieve the equilibration state earlier, as shown in Figure 3. It is suggested that the electrode hybridized by graphene induces much more GOx to catalyze the electrochemical process. As a result, the response current and the detection range increase. The electrocatalytic property of the modified electrode meets Menten kinetics and the apparent-Menten constant (*K_m_*) used to evaluate the affinity between enzyme and substrate. The higher the value of *K_m_*, the weaker the affinity, and vice versa. *K_m_* can be calculated by the Lineweaver–Burk equation:

(1) $$\frac{1}{I_{ss}}=\frac{K_{M}^{app}}{I_{max}}\times \frac{1}{C}+\frac{1}{I_{max}}$$

where *I_ss_* is the steady-state current with the substrate, *C* is the concentration of the substrate in solution and *I_max_* is the current when the substrate saturates. The apparent-Menten constants of GOx for the two electrodes are 7.18 and 15.64 mM, calculated by the above equation. The constant is in agreement with that reported in the literature [24], indicating that the composite film on the surface of the GOx/CNT-GO/PAA-Fc electrode has a stronger biological affinity and higher catalytic activity than that of electrode modified by CNTs only. The low *K_m_* value indicates that the immobilized enzyme retains its activity and that there is a low diffusion barrier.

### 3.4. Electron Transport on the Surface of the Electrode

**Figure 4:**
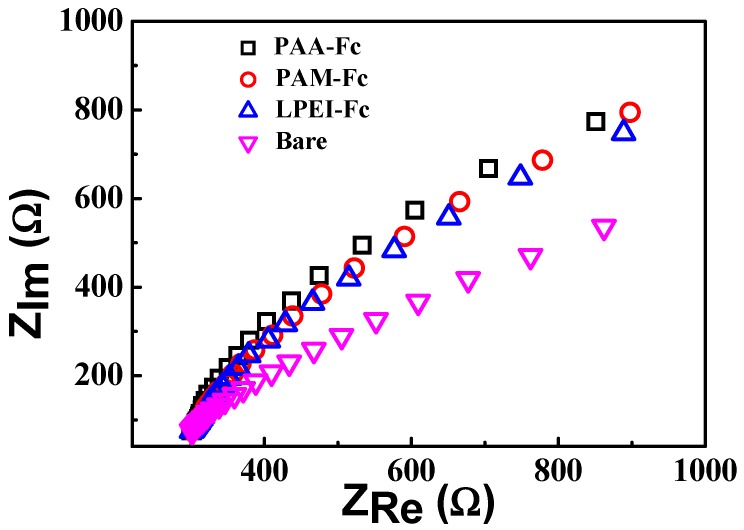
Nyquist plots corresponding to the impedance spectra for the three types of sensors (0.25 V vs. SCE, 100 mHz to 100 kHz).

The electron transfer in the enzyme electrode has been explored previously [25,26]. However, the factors that facilitate or impede electron transfer between the enzyme and electrode are still unclear. To clarify the possible role of redox polymer in the process of electron transfer, electrochemical impedance spectroscopy (EIS) was used to investigate the various redox polymer materials, and the results are shown in Figure 4. In addition, the results of saturated calomel electrode (SCE) are also presented as a reference. Electrochemical impedance spectroscopy can tell us about the impedance variation of the surface of the electrode. According to electrochemical theory, the semicircle at high frequency generally presents the impedance transferred by the electron, and the linear part at low frequency corresponds to the diffusion process of the substrate. As shown in Figure 4, the electron transfer impedances of the three polymer-modified electrodes are almost the same at high frequency, indicating that all these polyelectrolytes could act as a good electron-transfer interface between the EIS probe and the electrode. Comparing these results with the bare electrode illustrates that the polymer-modified electrode does not reduce the electron transfer efficiency between the electroactive sites embedded in the enzymes and the electrode. In addition, the resistance increases slightly after the polyelectrolyte is coated on the bare electrode, suggesting that the polyelectrolyte film blocks the electron exchange between the redox probe and electrode surface, but the diffusion rate does not reduce by much. The EIS results indicate that the three redox polymers can participate in the entire electron transfer process of GOx.

### 3.5. Sensitivity and Stability of the Biosensor

**Figure 5:**
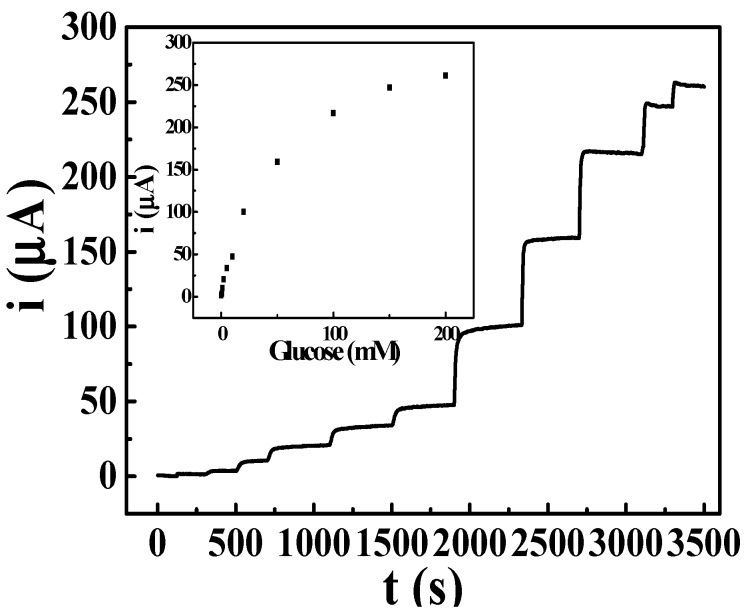
Typical amperometric response of the enzyme electrode following the successive injection of different amounts of glucose. Inset: the linear plot of current vs. glucose concentration.

Based on the voltammetric results described above, the amperometric current–time response of the GOx/CNT-GO/PAA-Fc was recorded with the successive addition of glucose in stirred PBS. The curve of the electric current of the enzyme electrode vs. glucose concentration is shown in the inset in Figure 5. A constant voltage with the value of −0.25 V was applied to the enzyme electrode. The glucose solution was then added into the cell several times as soon as the current began to stabilize. As indicated in Figure 5, the peak current increases significantly as more glucose solution is added. Thus, glucose plays a bioelectrocatalytic oxidation role in the enzyme electrode sensitively and quickly. In general, about 95% of the maximal response value can be reached within 10 s. In this work, the grafted ferrocenyl groups on the polymer backbone approached the redox centers of glucose oxidase. Consequently, the enzyme electrode acted more sensitively and quickly. Moreover, the enzyme electrode retained a good linear relationship with the glucose within a low concentration range, namely from 0.1 to 10 mM. Based on the above beneficial properties, the electrode can be used for the quantitative detection of glucose.

The current–time curve can be used to analyze the performance of different modified electrodes under the constant potential 0.5V. As shown in Figure S9, the current responses are rapid when glucose is added to the test solution and it reaches a steady state (95% of the maximum value) within 10 s, indicating that the biosensor possessed high sensitivity to glucose. Furthermore, Figure S9b shows the current–time curves of the PAA-Fc electrode at different concentrations of glucose. It can be seen that the PAA-Fc electrode shows good sensitivity in the concentration range investigated.

The operational and storage stability are vital for the application of the electrodes. The operational stability of the sensor can be evaluated by observing the change of current responses towards glucose using amperometry. The storage stability of the electrodes was also examined by comparing the changes of the current response at different times. After the electrodes were placed in PBS and refrigerated at 4 °C for six days, the current response retained 90% of the initial value with no obvious decline in the current response signal during the measurement, which suggests good stability of the prepared biosensor. To our knowledge, no report has used this type of polyelectrolyte film-coated GC electrode to selectively detect glucose up to now.

## 4. Conclusions

In the present work, the electrochemical properties of a GC electrode coated with a ferrocene-modified polyelectrolyte film were investigated. It was found that a GCE that combines the features of ferrocene-modified polyelectrolyte film with the properties of carbon nanomaterials enables electrochemical oxidations without an additional supporting electrolyte. The composite can be reused in subsequent electrolysis. In addition, the composite electrolyte can improve the kinetic property through the modification of the electrode surface. The prepared biosensor showed a wide linear range from 0.2 to 5 mM with a detection limit of 0.2 mM. The response time of the sensor reached 95% of the steady-state current in less than 10 s, which is indicative of a fast response. The electrode also displayed high operational stability and long-term storage stability.

## Supplementary Materials

The following are available online at https://www.mdpi.com/2073-4360/11/3/551/s1, Scheme S1: Schematics for the preparation of PAA-Fc; Scheme S2. Schematics for the preparation of PEI-Fc; Scheme S3. Synthetic protocol for PAM-Fc with ferrocene butyryl chloride modification; Scheme S4. Protocol for the enzyme electrode preparation; Figure S5. Cyclic voltammograms of different concentrations of PAA-Fc in PBS solution under a 0.1 V/s scan rate. Inset: Plot of the oxidation peak currents vs. the concentration of PAA-Fc; Figure S6. Cyclic voltammograms of 1% PAM-Fc and PEI-Fc in PBS solution; Figure S7. Cyclic voltammograms of blank electrodes without polyelectrolyte under different glucose concentrations; Figure S8. Cyclic voltammograms of the GOx/CNT/PAA-Fc and GOx/CNT-GO/PAA-Fc composite film in the absence (a, b) and presence (a’, b’) of 3.0 mmol/L glucose in PBS (pH = 7.2) at 100 mV/s; Figure S9. Performance of different modified electrodes (response to 5 mM glucose) and different concentrations of glucose (using the PAA-Fc electrode).
