# Supplementary material for: Ferrocene-Modified Polyelectrolyte Film-Coated Electrode and Its Application in Glucose Detection
Source: Polymers (Basel). 2019 Mar 22;11(3):551. doi: 10.3390/polym11030551 (PMC6473894; doi:10.3390/polym11030551)
Supplement: Supplementary file 1 [file polymers-11-00551-s001.pdf]

Supporting Information

Ferrocene-modified Polyelectrolyte Film coated Electrode and its Application in Glucose Detection

Yonggang SHANGGUAN [*], Zhiping JIANG, Qiang ZHENG

MOE Key Laboratory of Macromolecular Synthesis and Functionalization, Department of Polymer Science and Engineering, Zhejiang University, Hangzhou 310027, People's Republic of China

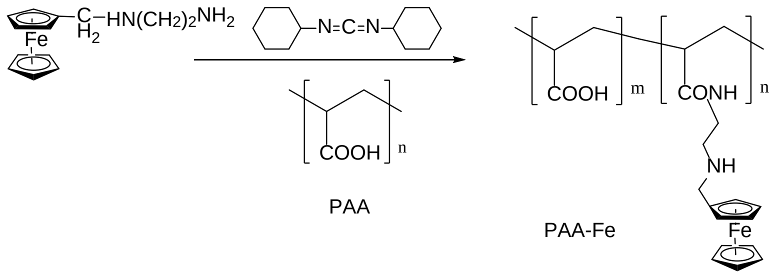

Scheme S1. Schematics for the preparation of PAA-Fc

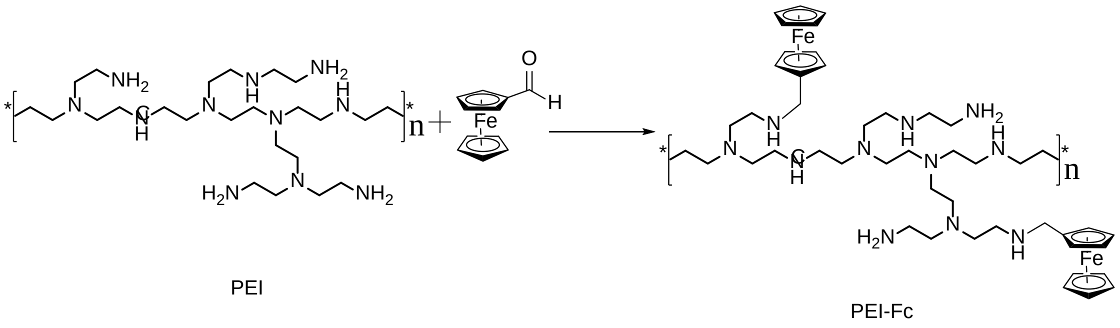

Scheme S2. Schematics for the preparation of PEI-Fc

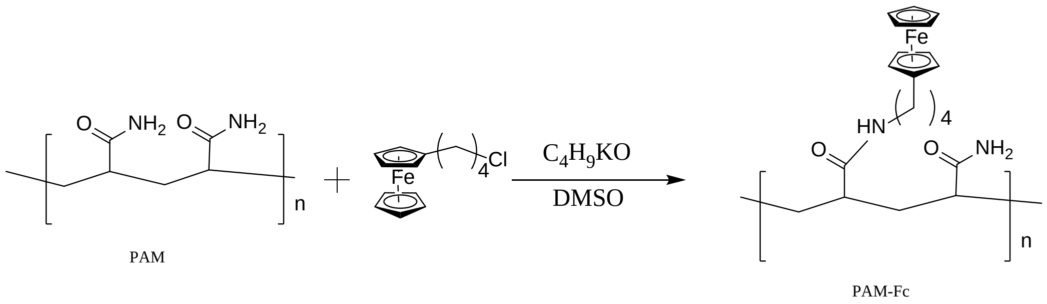

Scheme S3. Synthetic Protocol for the PAM-Fc with Ferrocene Butyryl Chloride Modification

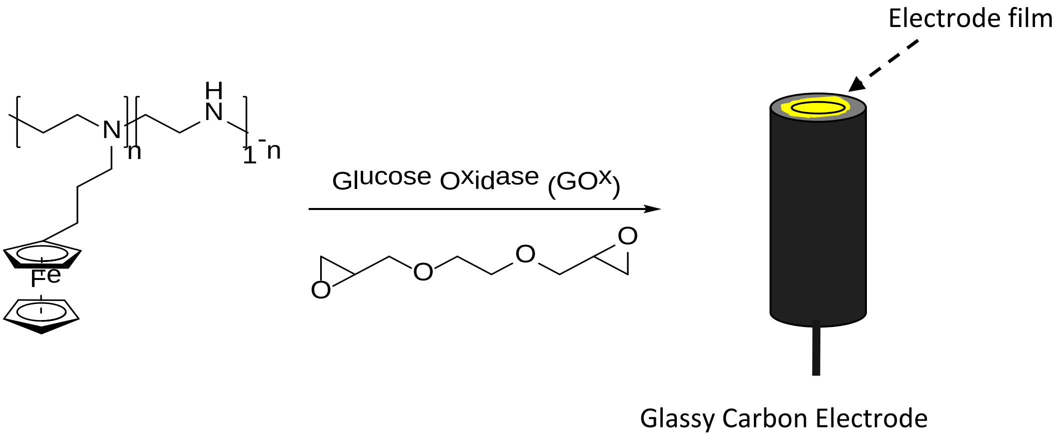

Scheme S4. Protocol for the Enzyme Electrode Preparation

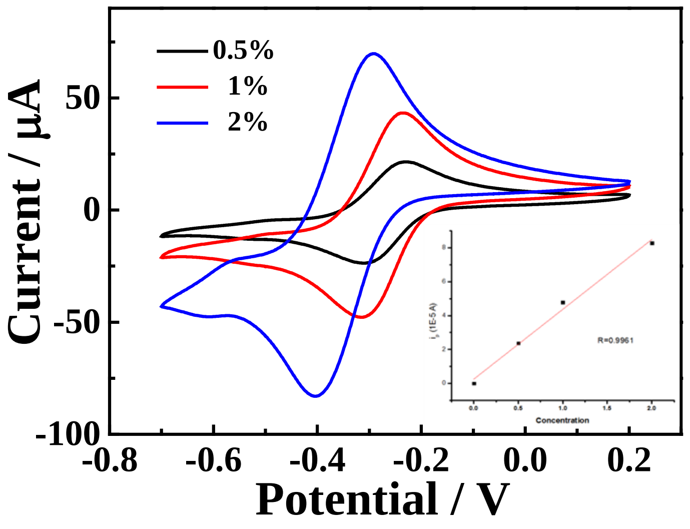

Fig. S5. Cyclic voltammograms of different concentration PAA-Fc in PBS solution under 0.1V/s scan rate. Inset: Plot of the oxidation peak currents vs. concentration of PAA-Fc

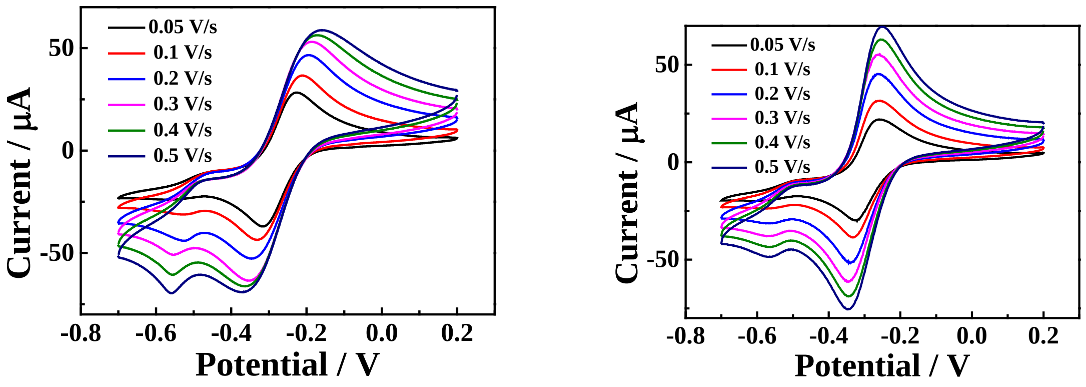

Fig. S6. Cyclic voltammograms of 1% PAM-Fc and PEI-Fc in PBS solution

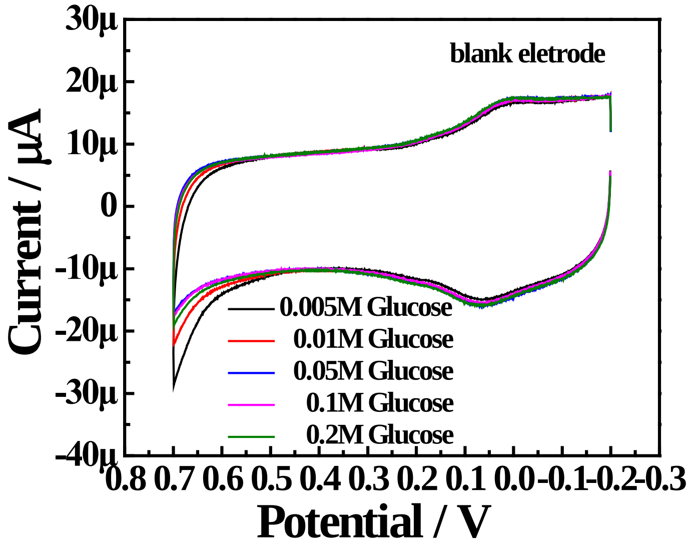

Fig. S7. Cyclic voltammograms of blank electrodes without polyelectrolyte under different glucose concentration

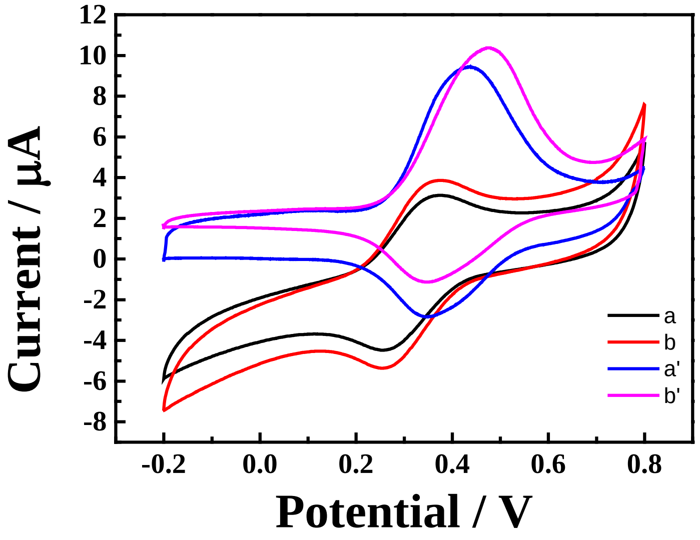

Fig. S8. Cyclic voltammograms of the GOx/CNT/PAA-Fc and GOx/CNT-GO/PAA-Fc composite film in the absence (a, b) and presence (a’, b’) of 3.0 mmol/L glucose in PBS (pH=7.2) at 100 mV/s

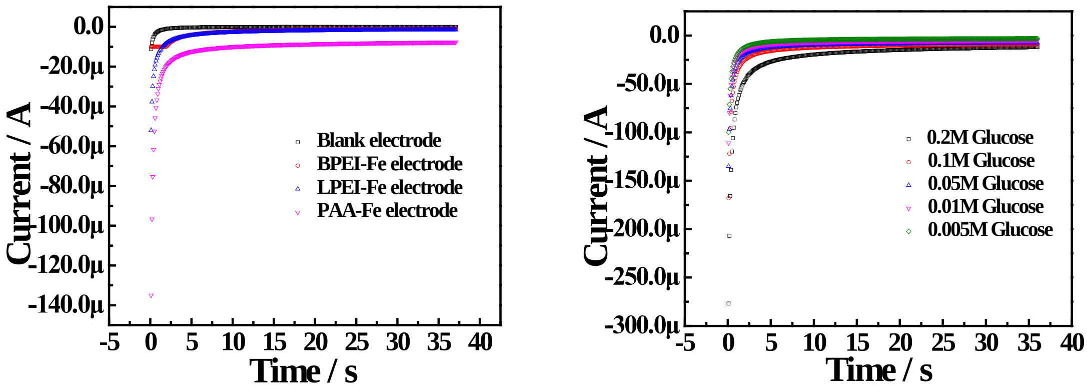

Fig. S9. Performance of different modified electrode (response for 5mM glucose) and different concentration of glucose (use PAA-Fc electrode)
